# Metropolitan Hospital Sunday Fund

**Published:** 1905-12-02

**Authors:** 


### METROPOLITAN HOSPITAL SUNDAY FUND.

### REPORT OF THE COUNCIL FOR 1905.

The Question of Grants to Nursing Associations.

The Lord Mayor (Alderman Vaughan Morgan) presided at a meeting of the Council of the Metropolitan Hospital Sunday Fund at the Mansion House on Tuesday, November 28.

Archdeacon Sinclair moved a resolution that the Distribution Committee be empowered to grant a certain amount of money to the three great London institutions for district nursing employing fully-trained hospital nurses. It had been shown, he said, that the work of nursing associations came well within the scope of the Fund. The question had come before the Committee, and certain objections had been mooted. The fear had been expressed that nursing associations might become an indefinite number, but the careful investigations which their Secretary had carried out had shown that the whole ground of London was covered by three well-known associations—Queen Victoria's Jubilee Institute, the North London Nursing Association, and the Bible Women and Nurses' Mission—all employing fully-trained hospital nurses. Other excellent institutions there were doing good work among the poor, but not coming within the scope of the resolution, since they did not employ trained nurses; and there need be no fear that they were supporting the amateur whims of amiable and unpractical ladies. The Queen Victoria Institute had 22 affiliated associations in London, employing 145 nurses, the districts of these associations covering nearly the whole of the Metropolitan area; 27 of these nurses were maintained almost entirely by the central body. The North London Association had 11 nurses, and the London District of the Bible Women and Nurses' Mission 81. They were engaged in relieving the sick poor in their own homes, and in the after treatment of hospital cases, so that their work was clearly akin to that of the hospitals themselves, and they worked under the same rules as were required of hospitals to whom grants were made; they were under the management of duly constituted committees, and they presented properly audited accounts.

The resolution was seconded by Mr. Deputy Wallace, and some discussion arose as to whether a limit should not be named of the amount to be allocated, as is done in the case of surgical appliances. It was contended that this might be done if thought necessary, by the annual meeting of constituents, but Sir Henry Burdett pointed out the inconvenience of thrashing out a question of detail in a big general meeting.

Mr. Norman suggested that instead of a fixed proportion of the amount collected by the Fund the sum allotted should be based on the needs of the institutions. On the same basis as that taken for the hospitals of about 10 per cent. of their needs, that would give these associations, the cost of whose nurses was £27,800, £2,800, while 5 per cent. of the £60,000 generally collected would be £3,000.

Mr. George Herring thought that it would place the Distribution Committee in an awkward position if some limit were not set.

Eventually the Archdeacon's resolution was put without amendment and carried *nem. con.*, as was also a motion by Sir Henry Burdett adding to it the provision that the amount allocated should not exceed 5 per cent. of the total collected.

On the motion of the Rev. E. H. Pearce a cordial expression of thanks to Sir Edmund Currie for the investigations he had made, and the information he had presented on the subject, was agreed to with acclamation.

### Hospitals and Medical Schools.

The report of the Council for the year ended October 31, 1905, was then presented. It stated that the thirty-third year of collecting the Fund had resulted, under the presidency of the Lord Mayor (Sir John Pound), the Treasurer, in a total of £78,379.

"The collections in the various places of worship resulted in an amount of £48,954, being, with the exception of 1903, when the Fund was augmented by the encouragement given by their Majesties' visit to St. Paul's, the largest on record.

"Mr. George Herring again added one-fourth to the amount collected in places of worship, and generously extended his gift to a special collection in the City, which resulted in an addition to the Fund of £1,298. His donation this year amounted to £12,400. Mr. William Herring and Mr. Charles Morrison each gave donations of £1,000; Sir Savile Crossley again divided his contribution of £1,000 between this Fund and the King's Fund, and "Delta" sent his twenty-seventh donation of £200.

"A legacy of £500 was paid by the executors of the late Mr. John Cohen.

"The editors of newspapers again most kindly and earnestly pleaded the urgent needs of the hospitals in the week preceding Hospital Sunday."

Mr. Charles Cheston, the Hon. Solicitor to the Fund, moved the addition to the Report of the following clauses: (1) This Committee cordially agrees with paragraph 27 of the report of Sir Edward Fry's Committee, appointed to inquire into the financial relation between the hospitals and medical schools, of which the following is an extract:—

> For the future the distinction between the hospital and the school should in every case be drawn, not only definitely and exactly, but with such clearness that it may be understood by the general public, and so that no question may arise as to the destination and application of moneys contributed, whether by the King's Fund or from any other source.

(2) No grant will be made from the Fund to any hospital which fails to completely comply with the above recommendation. (3) In estimating the amount to be awarded to the funds of any hospital from the Hospital Sunday Fund no moneys paid or subscribed during the preceding year towards the school attached to any hospital shall be taken into account.

With these additions the report was adopted and ordered to be published.

The following names were suggested to be put forward to the meeting of constituents to fill vacancies on the Council—namely, the Archdeacon of Middlesex, the Rev. R. J. Campbell, Sir Frederick Cook, Sir Frederick Treves, and the Rev. Charles Brown.

The Secretary announced that the next annual meeting of the constituents was fixed for Monday, December 18, and that the General Purposes Committee recommended that June 17, 1906, be fixed for next Hospital Sunday; and votes of thanks brought the proceedings to a close.

